# Controlled Release of Vancomycin and Tigecycline from an Orthopaedic Implant Coating Prevents *Staphylococcus aureus* Infection in an Open Fracture Animal Model

**DOI:** 10.1155/2019/1638508

**Published:** 2019-12-12

**Authors:** A. I. Stavrakis, S. Zhu, A. H. Loftin, X. Weixian, J. Niska, V. Hegde, T. Segura, N. M. Bernthal

**Affiliations:** ^1^Department of Orthopaedic Surgery, Orthopaedic Hospital Research Center, David Geffen School of Medicine at University of California Los Angeles, Los Angeles, CA, USA; ^2^Department of Chemical and Biomolecular Engineering, University of California Los Angeles, Los Angeles, CA, USA

## Abstract

**Introduction:**

Treatment of open fractures routinely involves multiple surgeries and delayed definitive fracture fixation because of concern for infection. If implants were made less susceptible to infection, a one-stage procedure with intramedullary nailing would be more feasible, which would reduce morbidity and improve outcomes.

**Methods:**

In this study, a novel open fracture mouse model was developed using *Staphylococcus aureus* (*S. aureus*) and single-stage intramedullary fixation. The model was used to evaluate whether implants coated with a novel “smart” polymer coating containing vancomycin or tigecycline would be colonized by bacteria in an open fracture model infected with *S. aureus*. *In vivo* bioluminescence, ex vivo CFUs, and X-ray images were evaluated over a 42-day postoperative period.

**Results:**

We found evidence of a markedly decreased bacterial burden with the local release of vancomycin and tigecycline from the PEG-PPS polymer compared to polymer alone. Vancomycin was released in a controlled fashion and maintained local drug concentrations above the minimum inhibition concentration for *S. aureus* for greater than 7 days postoperatively. Bacteria were reduced 139-fold from implants containing vancomycin and undetected from the bone and soft tissue. Tigecycline coatings led to a 5991-fold reduction in bacteria isolated from bone and soft tissue and 15-fold reduction on the implants compared to polymer alone. Antibiotic coatings also prevented osteomyelitis and implant loosening as observed on X-ray.

**Conclusion:**

Vancomycin and tigecycline can be encapsulated in a polymer coating and released over time to maintain therapeutic levels during the perioperative period. Our results suggest that antibiotic coatings can be used to prevent implant infection and osteomyelitis in the setting of open fracture. This novel open fracture mouse model can be used as a powerful *in vivo* preclinical tool to evaluate and optimize the treatment of open fractures before further studies in humans.

## 1. Introduction

Open fractures are often devastating injuries for patients. In addition to challenges of fracture healing in dramatically injured and denudated bone, exposure to the “outside world” results in osteomyelitis in up to 50% of cases [1, 2]. Nonunion rates are reported as high as 16% in open fractures (as compared to less than 5% in closed fractures), and eventual amputation is not uncommon [3, 4]. Even with successful treatment, patient satisfaction scores are low and the cost to treat these infections is high [5, 6]. Annually, there are approximately 6 million fractures in the United States, and nearly 4% are open, suggesting an estimated 240,000 open fractures [5, 7]. This epidemic is particularly pronounced in the military, where contaminated extremity fractures from blast wounds remain the most common injury of military personnel in the battlefield with associated infection rates reported as high as 20–30% [8–10]. The standard of care for open fractures includes administration of intravenous antibiotics as soon as possible after the injury, urgent washout, and continuation of antibiotics for a minimum of 24 hours after wound closure [11]. Surgical fixation of open fractures depends on the severity and location of the injury. While most long bone injuries are treated with intramedullary nail fixation, periarticular or small bone fractures are often treated with plates and screws, and external fixation remains a viable treatment option, especially for temporization.

Infection rates remain high despite this “best practice” [5, 6]. This is partially due to the formation of biofilm on the implant surface, which prevents intravenous antibiotics and the innate immune response from reaching bacteria [12, 13]. While one option to decrease this phenomenon is to perform repeat irrigation and debridement procedures to clean the fracture site prior to internal fixation, this requires multiple surgeries, a prolonged hospital stay, and an enormous cost to the healthcare system. Another option is to utilize external fixation, but patient satisfaction scores are low and repeat operation for definitive fixation is usually required [14]. Therefore, new methods must be developed to allow efficient orthopaedic stabilization of fractures in the setting of contaminated wounds. A one-step method of fracture fixation with a device that both protects its own surface from bacterial adhesion and releases antibiotics to improve local clearance of infection has potential to improve patient outcomes and conserve resources. The concept of local delivery of antibiotics in the setting of an open fracture is not a new one. Antibiotic irrigation has been used for many years in open fractures although recent literature has questioned its effectiveness [6, 15]. Vancomycin and gentamicin have been delivered locally in open fractures using polymethyl methacrylate (PMMA) cement beads, resulting in lower postoperative infection rates compared to intravenous antibiotics alone (4% versus 12%–16%) [16, 17]. However, these patients require a second operation to remove the residual cement, and the implant surface is not protected, leaving it susceptible to bacterial seeding and biofilm formation. Newer biodegradable antibiotic beads have been developed, eliminating the need for a second operation, but they also do not protect the implant from bacterial seeding or elute antibiotics into the intramedullary canal to prevent ascending or descending osteomyelitis. Antibiotic cement has also been used to coat intramedullary implants [18]. Drawbacks of this technique include limited control of elution properties, long-term retention of a nonantimicrobial coating once the antibiotics have eluted, and the inability to coat plates and screws. Our objective in this study was to develop a bioabsorbable implant coating to deliver antibiotics locally via an intramedullary implant. Additionally, the polymer coating was designed to confer several specific unique benefits: (1) the coating polymerizes rapidly in a nonexothermic reaction, allowing incorporation of heat sensitive antibiotics and antimicrobials, (2) the coating can release antibiotics over a sustained period of time, and (3) the coating is completely biodegradable and has been demonstrated to not have a negative effect on osseointegration [19]. A mouse model of postarthroplasty infection using a bioluminescent strain of *Staphylococcus aureus* (*S. aureus)* has previously been established [20–24]. This model has been used to understand the immune response to such infections as well as to the evaluate the efficacy of perioperative antibiotics and treatment of chronic infections [22, 24]. In the present study, we adapted our mouse model to an open fracture model by creating a defect in the femoral diaphysis and inoculating *S. aureus* into the open thigh wound, more consistent with a blast injury. Vancomycin is commonly used in the treatment of such open fracture infections, but it is unclear if it is the optimal antibiotic against *S. aureus* [25]. Tigecycline is a newer antibiotic with increased antimicrobial activity against both MSSA and MRSA, especially against biofilm formation in both *in vitro* and *in vivo* settings [26, 27]. Thus, we incorporated vancomycin or tigecycline into a novel PEG-PPS polymer covalently linked to an intramedullary implant and evaluated the efficacy of these coatings against a local *S. aureus* infection.

## 2. Methods

### 2.1. Synthesis of PEG-PPS Block Copolymer Antibiotic Coatings

The synthesis of the PEG-PPS polymer was the same as previously described [19]. An orthopaedic-grade titanium Kirschner-wire (K-wire) (diameter 0.6 mm) underwent oxygen plasma treatment at 200 mTorr and 200 W for 15 min. Subsequently, 1% (v/v) (3-mercaptopropyl) trimethoxysilane was reacted with the K-wire in toluene at 90°C with stirring followed by sonication in chloroform (5 times), acetone (2 times), methanol (5 times), and milliQ water (5 times). The K-wire was then heated at 50°C for at least 30 minutes to dry. The PEG-PPS polymer was then dissolved in phosphate buffer saline (PBS) to make either a 3% or 6% (w/v) solution, which was used to dissolve either tigecycline or vancomycin at a concentration of 20 mg/ml. The K-wires were submerged in the antibiotic-encapsulated PEG-PPS solution or PEG-PPS solution alone at 4°C and dried at 50°C. This wet-dry cycle was repeated for a total of ten times. To aid in visualization of the coating, rhodamine was also coated on the titanium pins via encapsulation in PEG-PPS solution. The microstructure of the polymer coatings was characterized using Nano SEM with X-ray microanalysis.

### 2.2. *In Vitro* Antibiotic Release Kinetics

*In vitro* release of vancomycin and tigecycline from the PEG-PPS coating on titanium K-wires was conducted, as previously described and validated, by submerging pins in 200 *μ*l of PBS at 37°C. The amount of released vancomycin was quantified by high-performance liquid chromatography (HPLC) based on its UV absorption at 280 nm, and the buffer was refreshed daily for at least one week. Known concentrations (0, 1, 5, 10, 20, 40, and 100 *μ*g/mL) of vancomycin dissolved in PBS were used to generate a calibration curve with HPLC under the same method, using 0.1% trifluoroacetic acid at a flow rate of 0.1 mL/min [19].

### 2.3. *S. aureus* Bioluminescent Strain

*S. aureus* Xen36 (PerkinElmer, Hopkinton, MA) is a bioluminescent derivative of the *S. aureus* ATCC 49525 (Wright) strain, a clinical isolate from a bacteremic patient that has been used in previous animal studies [20, 24, 28]. This strain has a Gram-positive optimized *luxABCDE* operon stably integrated into a native plasmid, and live, actively metabolizing bacteria naturally emitting a blue-green light with a maximal emission wavelength of approximately 490 nm (Francis 2000). The minimum inhibition concentration for Xen36 for vancomycin is ≤0.5 *μ*g/ml and for tigecycline is ≤0.25 *μ*g/ml. *S. aureus* Xen36 has previously been shown to be optimal for use due to the strength and consistency of its signal [20].

### 2.4. Preparation of *S. aureus* for Inoculation

*S. aureus* Xen36 possesses a kanamycin resistance selection marker, enabling isolation from contaminating background strains during culture. Thus, 200 *μ*g/ml kanamycin (Sigma-Aldrich, St. Louis, MO) was added to all cultures. *S. aureus* Xen36 was streaked onto tryptic soy agar plates (tryptic soy broth (TSB) plus 1.5% bacto agar (BD Biosciences, Franklin Lakes, NJ)) and grown at 37°C overnight [20–24]. Single colonies were cultured in TSB and grown overnight at 37°C in a shaking incubator (240 rpm) (MaxQ 4450; Thermo, Waltham, MA). Midlogarithmic phase bacteria were obtained after a 2 h subculture of a 1 : 50 dilution of the overnight culture. Cells were pelleted, resuspended, and washed 3 times in phosphate buffered saline (PBS). Bacterial concentrations were estimated by measuring the absorbance at 600 nm (A600; Biomate 3 (Thermo, Waltham, MA)). Colony-forming units (CFUs) were verified after overnight culture of plates.

### 2.5. Mouse Surgical Procedures

Twelve-week-old male C57BL/6 mice (*n* = 6 per group) were used in all experiments. Animals were kept at 3 mice per cage in standard cages with a 12-hour light and dark cycle. They were fed a standard pellet diet with free access to bottled water. Assessments were carried out daily by veterinary staff to ensure the well-being of all animals throughout the experiment. The surgical procedure for this open fracture model was adapted from the surgical procedure from our previously validated mouse model of prosthetic joint infection [20–24]. A medial parapatellar knee arthrotomy was made and a medical-grade titanium K-wire coated with either PEG-PPS alone, PEG-PPS containing vancomycin, or PEG-PPS containing tigecycline was implanted into the femoral intramedullary canal in a retrograde fashion. A separate incision was then made 1 cm proximal to the joint space on the lateral aspect of the thigh and a 2 mm femoral defect created at the level of the midshaft femur. A micropipette was used to inoculate *S. aureus* Xen36 (1 × 108 CFUs in 2 *μ*l saline) into the surgically created open fracture site. The two surgical wounds were closed using 5–0 vicryl sutures. Analgesia (2.5 mg/kg sustained-release buprenorphine) was administered at the time of surgery as well as every 3 days postoperatively.

### 2.6. High-Resolution X-Ray Imaging

Lateral radiographs of the femur were obtained using high-resolution X-ray (Faxitron LX-60 DC-12 imaging system) immediately after surgery to ensure proper placement of the implant. Images were also taken on postoperative day (POD) 21 and POD 42. A fellowship trained orthopaedic surgeon evaluated the high-resolution X-ray images to determine whether vancomycin- or tigecycline-coated implants had any impact on bony architecture and implant stability at 3 and 6 weeks. The evaluator was blinded to the treatment groups and assessed implant stability, osteolysis, and appearance of osteomyelitis or involucrum.

### 2.7. Quantification of *S. aureus* Bacterial Burden Using Bioluminescence Imaging *In Vivo* and CFUs

Mice were anesthetized via inhalation of isoflurane (2%) and *in vivo* bioluminescence imaging was performed by using the Xenogen *in vivo* imaging system (Xenogen IVIS; Caliper Life Sciences, Waltham MA). Images were obtained on POD 0, 3, 7, 14, 21, 28, 35, and 42 as previously described [20–24]. Data are presented on color scale overlaid on a grayscale photograph of mice and quantified as maximum flux (photons per second (s) per cm^2^ per steradian (sr) (p/s/cm^2^/sr)) within a circular region of interest (1 × 103 pixels) by using Living Image software (Xenogen, Alameda, CA).

To confirm that the bioluminescence signals corresponded to the bacterial burden *in vivo*, the mice were euthanized following the last day of imaging, POD 42, and the implant and surrounding tissue were cultured. Bacteria were detached from the implant by sonication in 1 ml 0.3% Tween-80 in TSB for 10 minutes followed by vortexing for 5 minutes as previously described [16]. In addition, bacteria in the surrounding joint tissue were measured by homogenizing bone and joint tissue (Pro200H Series homogenizer; Pro Scientific, Oxford, CT). The number of bacterial CFUs that were adherent to the implant and in the joint tissue was determined by counting CFUs after overnight culture of plates and was expressed as total CFUs harvested from the implant and joint tissue. To confirm the absence of bacteria in samples measuring zero CFUs, homogenates were cultured in TSB for 48 hours at 37°C in a shaking incubator and then plated overnight, and presence or absence of CFUs was determined.

### 2.8. Statistical Analysis

Each group had 6 mice based on previous bioluminescence studies from our group showing that 6 animals/group were necessary to attain statistical significance [16–18, 20, 21]. Data between two groups were compared using Student's *t*-test (one- or two-tailed where indicated), while data between three or more groups were compared using a one-way ANOVA. All data are expressed as mean and standard error of the mean (SEM). Values of *p* < 0.05 were considered statistically significant.

## 3. Results

### 3.1. Release Kinetics of Vancomycin and Tigecycline from PEG-PPS Coatings

**Figure 1:**
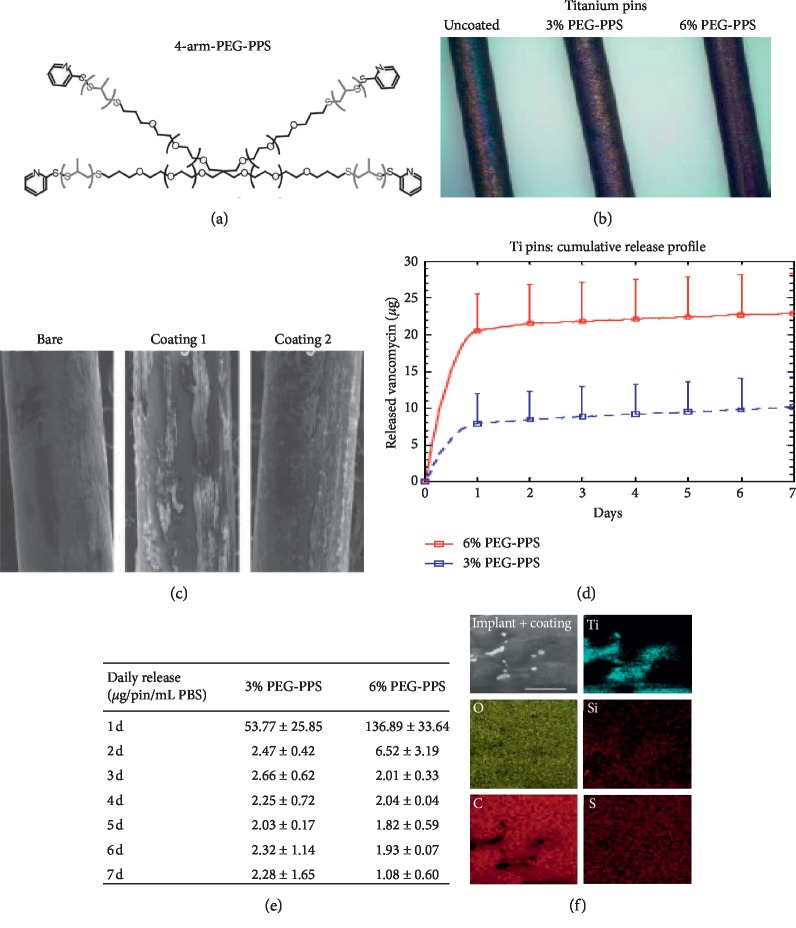
(a) The structure of PEG-PPS polymer used to coat metal implants. (b) The visualization of the polymer coating is demonstrated by a model molecule, rhodamine, which was dispersed in the PEG-PPS coating solution prior to coating (−, bare titanium pin; +3(w/v)% PEG-PPS-coated pin containing rhodamine; ++, 6(w/v) % PEG-PPS-coated pin containing rhodamine). (c) Scanning electron microscopic (SEM) images of the surfaces of a bare titanium pin, a titanium pin-coated with PEG-PPS containing vancomycin, and a titanium pin-coated with PEG-PPS containing tigecycline. (d) *In vitro* release of vancomycin from 3 to 6 (w/v)% of PEG-PPS-coated titanium K-wires in 150 *μ*l of PBS at 37°C. (e) Daily release vancomycin per pin quantified via HPLC (*N* = 3, mean ± SEM). (f) A higher magnification of the surface morphology and the X-ray microanalysis of available elements on the surface of a PEG-PPS-coated titanium pin.

The titanium pins were visually examined to be red on the surface, confirming application of antibiotic coating with use of rhodamine (Figure 1). SEM was used to evaluate the microstructure of the polymer and demonstrated 97% coverage with a smooth polymer surface and coating thickness on the micron scale (Figure 1). Pins were submerged in PBS, and the *in vitro* release of vancomycin and tigecycline was determined using HPLC, demonstrating the same burst pattern as previously described [19].

### 3.2. Effect of Antibiotic Coatings on *In Vivo* Bioluminescent Signals

**Figure 2:**
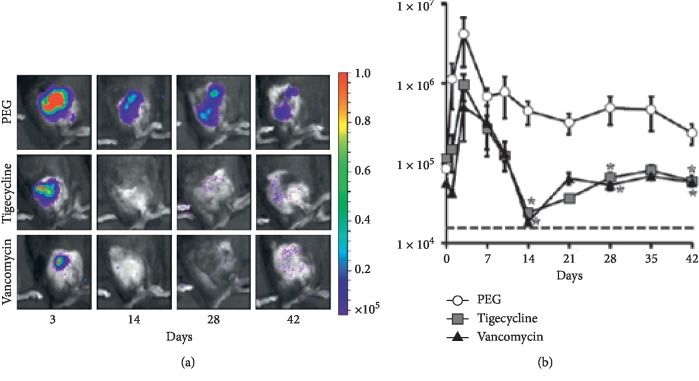
(a) *In vivo* bioluminescence images of a representative mouse from each group over the study period. (b) Mean *in vivo* bioluminescence signals throughout the study period.

Bacterial burden, as measured by bioluminescence signaling, peaked on day 7 (5.7 × 106 ± 7.4 × 106 photons/s/cm^2^/sr) in the mice treated with PEG-PPS alone and remained above 2.4 × 5 photons/s/cm^2^/sr throughout the 42-day experiment, indicating a chronic infection (Figures 2(a) and 2(b)). Vancomycin coatings resulted in a statistically significant reduction in bioluminescence signals on days 14, 28, and 42 (25-, 9-, and 4-fold, respectively, *p* < 0.01). Tigecycline coatings also resulted in a statistically significant reduction in bioluminescence signals on days 14, 28, and 42 (19-, 8-, and 4-fold, respectively, *p* < 0.01) compared with PEG-PPS alone. There was no difference in bacterial burden between vancomycin and tigecycline treatment groups throughout the 42-day study period.

### 3.3. Effect of Antibiotic Coatings on Ex Vivo Bacterial Counts

**Figure 3:**
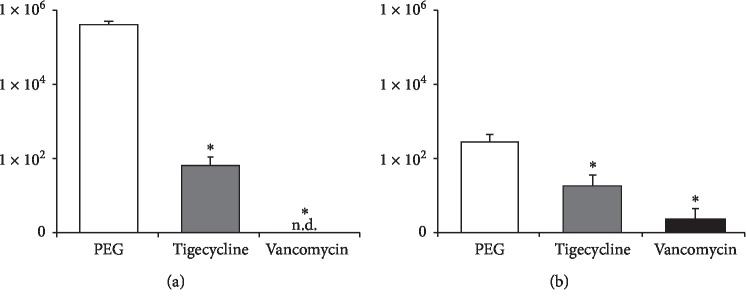
(a) CFU harvested from the joint tissue (log scale). (b) CFU harvested from the implants (log scale).

Implants and surrounding soft tissue and bone from the fracture site were harvested at the completion of the experiment (POD 42) and ex vivo CFUs determined (Figures 3(a) and 3(b)). Mice implanted with PEG-PPS alone had 3.7 ± 1.1 × 105 CFUs isolated from the peri-implant tissue and 2.8 ± 1.5 × 102 CFUs isolated from the implants. Vancomycin- and tigecycline-coated pins both resulted in a significant reduction in bacterial CFUs after the 42-day experiment. Vancomycin-coated pins grew out a mean of 2 ± 2 CFUs (139-fold reduction) and zero CFUs in the peri-implant tissue. Tigecycline-coated pins resulted in a mean of 1.8 ± 1.8 × 101 CFUs from the implants (15-fold reduction) and 6.3 ± 4.5 × 101 CFUs isolated from the peri-implant tissue (5991-fold reduction). The homogenized samples were then cultured for an additional 48 hours to confirm the presence or absence of bacteria. Vancomycin coatings resulted in bacteria present in 1 of 5 implant cultures and 1 of 5 peri-implant tissue cultures. The tigecycline group had bacteria present in 2 of 5 of the ex vivo peri-implant tissue cultures and 1 of 5 of the ex vivo implant cultures.

### 3.4. Effect of Antibiotic Coatings on X-Ray Imaging

**Figure 4:**
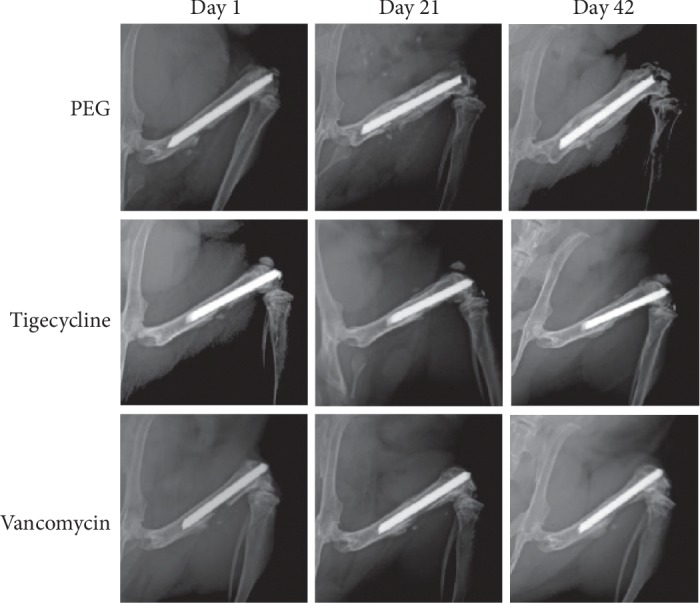
Representative radiograph from a mouse in each group over the study period.

Mice treated with PEG-PPS alone, via qualitative analysis, displayed more radiographic findings consistent with osteolysis, bony destruction, involucrum, and implant loosening, when compared to mice treated with vancomycin or tigecycline (Figure 4). The high-resolution X-rays demonstrated qualitatively that both vancomycin and tigecycline coatings prevent radiologic changes observed in the setting of implant infection and osteomyelitis.

## 4. Discussion

The most important finding of the present study is that antibiotic coatings can be used to prevent implant infection and osteomyelitis in an open fracture setting. Open fractures have a higher incidence of infection, nonunion, and adverse outcomes and generally lead to decreased patient satisfaction and increased healthcare costs [6]. Decreasing the rate of infection could increase the likelihood of fracture healing and improve outcomes. Preclinical data have demonstrated efficacy of tigecycline against *S. aureus* in rabbit and rat models of osteomyelitis [29, 30]. Thus, we compared tigecycline to vancomycin in a novel antibiotic coating applied to an intramedullary implant in an open fracture model. To our knowledge, this is the first open fracture model in mice using intramedullary implants, and this model allows us to evaluate potential therapeutic interventions for open fractures in a short time period and obtain multiple endpoints in a single mouse. Several key findings are presented in this study. First, we demonstrated that vancomycin and tigecycline can be encapsulated in a PEG-PPS polymer covalently linked to implants and released for more than 7 days to maintain therapeutic levels during the perioperative period. Second, local release of vancomycin and tigecycline from the PEG-PPS coatings resulted in significant reductions in bacterial burden on the implant surface as well as in the surrounding bone and soft tissue. Third, bacteria were only present on one in five implants coated with vancomycin and one in five implants coated with tigecycline, as compared to all five in uncoated and polymer-coated alone groups. Finally, the addition of vancomycin or tigecycline to the coatings prevented bony changes seen on X-ray in the setting of chronic implant infection and osteomyelitis. Prior studies evaluating the efficacy of local antibiotics have focused on vancomycin-, gentamicin-, or tobramycin-loaded cement beads [16, 17, 31]. These studies found a significant decrease in infection rates when local antibiotics were used in addition to the standard of care (washout, debridement, and at least 24 hours of intravenous antibiotics) [16, 17, 31]. More recent animal studies have evaluated bioabsorbable antibiotic elution vehicles such as demineralized bone matrix, calcium sulfate, and fibrin clots [32, 33]. One such study in goats found tobramycin-impregnated calcium sulfate pellets were effective at preventing *S. aureus* infection in an infected proximal tibial defect [32]. Our coating is unique because it has an inner layer of polymer covalently linked to the implant, which makes it resistant to wear and shear forces during implantation, and an outer polymer layer which has an active release of antibiotics driven by reactive oxygen species (induced by neutrophil influx during infection) and a passive release that maintains antibiotic levels above the MIC for one week after surgery (in the vancomycin group). These properties protect the implant from biofilm formation and enable the delivery of antibiotics within the medullary canal as well as the surrounding soft tissues. Preventing biofilm formation is critically important because once a biofilm is formed, it is nearly impossible to clear the infection without removing the implant. Current methods of antibiotic delivery (antibiotic cement and biodegradable antibiotic beads) “clean the neighborhood” but do not protect the surface of the implant. In our study, 8 of 10 antibiotic-coated implants were protected from bacterial biofilm formation based on 48-hour culture in broth on a shaking incubator.

Another specific advantage of a polymer coating on intramedullary implants such as a tibial nail for an open tibia fracture as opposed to using a cement-coated implant is that the polymer coating maximizes the intramedullary nail diameter. Antibiotic cement can be used to coat intramedullary nails; however, the nail diameter must be downsized in order to accommodate the added cross-sectional area that a cement coating creates [18].

Although cement-coated intramedullary nails can improve infection rates, smaller diameter nails have a higher rate of failure and can delay progression of weight bearing [18]. Therefore, a low-profile polymeric coating could enable surgeons to use a larger diameter nail, potentially allowing earlier weight bearing and resulting in lower failure rates. In addition, our polymer coating can be applied to plates and screws, expanding the reach of this technology beyond the current scope using antibiotic cement and biodegradable beads.

In our study, delivery of local vancomycin and tigecycline preserved bone architecture and enabled fracture healing as observed on X-ray. One limitation of our study is that we did not look more extensively at other endpoints of fracture healing, such as micro-CT or histology. Although fracture union is an important endpoint and necessary to translate these findings into clinical practice, it is beyond the scope of this study since we are presenting proof of concept for a novel open fracture model and implant coating with controlled release of antibiotics. To further demonstrate efficacy of this coating, we will assess the impact of the antibiotic coating on new bone formation, bone remodeling, and osseointegration as determined through manual testing, histology, and micro-CT.

Elution of antibiotics from PMMA beads has been shown to result in high local concentrations in the bone and soft tissue, while limiting serum concentrations [33]. This allows for maximal local effect, while minimizing systemic toxicity. Although we did not specifically measure serum concentrations in this study, it can be assumed that local release of antibiotics in our study results in similar systemic uptake as previously described [34]. A high local concentration and low serum concentration is especially important for tigecycline, which commonly causes nausea and vomiting (seen in 1 of 4 patients) when used intravenously. These side effects are dose dependent. Therefore, local release of tigecycline could be used to effectively treat open fractures or chronic infection while limiting side effects [35, 36]. Interestingly, the concentration of tigecycline released from the polymer coatings in this study was below the MIC for Xen36 *S. aureus* but was effective in eliminating bacteria in 3 of 5 mice. This may be due to the large initial burst of tigecycline on day 1 or theoretically because local antibiotic therapy requires lower concentrations of drug to achieve an antimicrobial effect.

As previously mentioned, we were unable to completely eliminate infection in our study, as 1 mouse in the vancomycin group and 2 in the tigecycline group had bacteria present on the implant or in the peri-implant tissue after the 42-day experiment. One explanation for this is that we used local antibiotic therapy alone without the addition of intravenous antibiotics or surgical washout, which is the standard of care for open fractures. We plan to assess the efficacy of combining intravenous antibiotics with antibiotic coatings in future studies. Another approach is to optimize the coating by incorporating a synergistic drug such as rifampin, which has enhanced antibiofilm activity and therapeutic benefit in cases of retained hardware [37–45].

In conclusion, the open fracture mouse model presented in this study demonstrated a therapeutic benefit of vancomycin- and tigecycline-coated implants against a local *S. aureus* infection. This study provides important preclinical evidence in support of a novel antibiotic coating to prevent biofilm formation and release antibiotics into bone and soft tissue to decrease infection associated with open fractures. In particular, this coating allows for one-stage fracture fixation without the need for a second surgery to remove the antibiotic delivery vehicle (i.e., cement beads), which has the potential to improve clinical outcomes. Taken together, our findings suggest that this open fracture mouse model could serve as a valuable preclinical *in vivo* model to evaluate and optimize the treatment of open fractures prior to further studies in humans.

## Data Availability

Original data for this study are archived in the Nicholas Bernthal Laboratory at the Orthopaedic Hospital Research Center at the University of California, Los Angeles.
